# Introducing *Candidatus* Bathyanammoxibiaceae, a family of bacteria with the anammox potential present in both marine and terrestrial environments

**DOI:** 10.1038/s43705-022-00125-4

**Published:** 2022-05-19

**Authors:** Rui Zhao, Jennifer F. Biddle, Steffen L. Jørgensen

**Affiliations:** 1 School of Marine Science and Policy, University of Delaware, Lewes, DE USA; 2 Centre for Deep Sea Research, Department of Earth Science, University of Bergen, Bergen, Norway; 3 Present Address: Department of Earth, Atmospheric and Planetary Sciences, Massachusetts Institute of Technology, Cambridge, MA USA

**Keywords:** Water microbiology, Biogeochemistry

## Abstract

Anaerobic ammonium oxidation (Anammox) bacteria are a group of extraordinary bacteria exerting a major impact on the global nitrogen cycle. Their phylogenetic breadth and diversity, however, are not well constrained. Here we describe a new, deep-branching family in the order of *Candidatus* Brocadiales, *Candidatus* Bathyanammoxibiaceae, members of which have genes encoding the key enzymes of the anammox metabolism. In marine sediment cores from the Arctic Mid-Ocean Ridge (AMOR), the presence of *Ca*. Bathyanammoxibiaceae was confined within the nitrate-ammonium transition zones with the counter gradients of nitrate and ammonium, coinciding with the predicted occurrence of the anammox process. *Ca*. Bathyanammoxibiaceae genomes encode the core genetic machinery for the anammox metabolism, including hydrazine synthase for converting nitric oxide and ammonium to hydrazine, and hydrazine dehydrogenase for hydrazine oxidation to dinitrogen gas, and hydroxylamine oxidoreductase for nitrite reduction to nitric oxide. Their occurrences assessed by genomes and 16S rRNA gene sequencings surveys indicate that they are present in both marine and terrestrial environments. By introducing the anammox potential of *Ca*. Bathyanammoxibiaceae and charactering their ideal niche in marine sediments, our findings suggest that the diversity and abundance of anammox bacteria may be higher than previously thought, and provide important insights on cultivating them in the future to not only assess their biogeochemical impacts but also constrain the emergence and evolutionary history of this functional guild on Earth.

## Introduction

Anammox bacteria play critical roles in the global nitrogen cycle, especially in nitrogen loss in natural [1–6] and engineered environments [7, 8]. Currently known anammox bacteria are affiliated to five different genera in two families in the order *Candidatus* Brocadiales [per the nomenclature of Genome Taxonomy Database (GTDB; [9])]: *Candidatus* Brocadia, *Candidatus* Kuenenia, *Candidatus* Anammoxoglobus and *Candidatus* Jettenia [10] in the family of *Candidatus* Brocadiaceae, and *Candidatus* Scalindua in the family *Candidatus* Scalinduaceae. Considering that the phylogenetic breadth of functional guilds has been greatly expanded due to the explosion of available genomic data, such as bacteria involved in sulfate reduction [11] and prokaryotes responsible for ammonia oxidation [12–15], it remains unclear whether the currently characterized anammox lineages can fully represent all potential anammox bacterial diversity.

There is a general partitioning of the anammox bacteria between the marine and terrestrial environments. Anammox bacteria in the marine environment are dominated by *Ca*. Scalindua [16–20]. In contrast, anammox bacteria in the diverse terrestrial habitats hosted the other four lineages [4, 21], although *Scalindua* were also occasionally noted in in engineered [22] and freshwater systems [23]. The underlying mechanisms driving this ecological niche partitioning are not fully elucidated yet [24]. Recovering more deeply branching lineages of anammox bacteria would be valuable to illuminate this ecophysiological division and evolutionary history of this important functional guild in geological history.

Here we report a deep-branching family within the order of *Ca*. Brocadiales, which harbor members from both marine and terrestrial environments. Members of this family have a confined distribution within the nitrate-ammonium transition zones (NATZs) of marine sediments where the anammox reaction was predicted to occur, and their genomes encode the key enzymes of the anammox metabolism. Our study suggests that the diversity, abundance, and ecological impact of anammox bacteria may be higher than previously thought. The newly recognized family of anammox bacteria is also valuable to delineate the emergence and evolutionary history of anammox bacteria in the geological history.

## Results and discussion

### An uncharted deep-branching family within the order Brocadiales

**Fig. 1:**
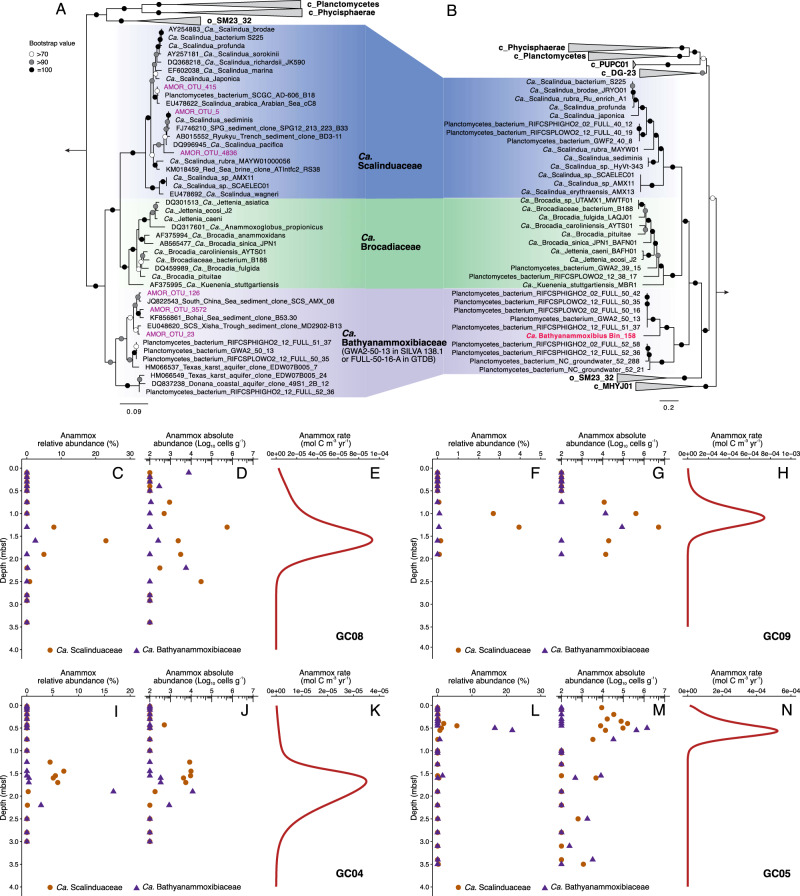
Phylogeny and distribution of anammox bacteria in four AMOR sediment cores. (**A**, **N**) Maximum-likelihood phylogenetic trees of bacteria in the order of Brocadiales based on the 16S rRNA gene (**A**) and 120 concatenated bacterial single-copy genes (**B**). The trees were inferred using IQ-TREE with GTR + F + R5 and LG + F + R7 as the best-fit evolutional model, and 1,000 times of ultrafast bootstrap iteration were applied to assess the robustness of both trees. The metagenome-assembled genome (MAG) recovered from the AMOR sediment is highlighted in red. The nomenclature used here following GTDB (https://gtdb.ecogenomic.org), while the new anammox family *Ca*. Bathyanammoxoceae (f__2-02-FULL-50-136 in GTDB 06-RS202 release) is named in this study. Bootstrap values of >70 are shown with symbols listed in the legend. The scale bars show estimated sequence substitutions per residue. **C**–**N** Vertical distribution of anammox bacteria and the predicted anammox reaction rates in AMOR sediment cores. The relative abundance of the two anammox families (**B**, **E**, **H**, **K**), *Ca*. Scalinduaceae and Ca. Bathyanammoxoceae, were determined by the sum of the OTUs assigned to these groups (showed in **A**), based on the 16S rRNA gene amplicon sequencing data reported in ref. [20]). The anammox reaction rates (**D**, **G**, **J**, **M**) were estimated by the reaction-transport model by ref. [20]. Panels (**C**–**N**) are modified from Fig. 2 of Ref. [20], an open access article distributed under Creative Commons Attribution License 4.0 (CC BY).

In the amplicon sequencing data of the four Arctic Mid-Ocean Ridge (AMOR) sediment cores (GC08, GC09, GC04, and GC05) reported in [20], there were three OTUs (OTU_23, OTU_126, and OTU_3572) that were classified as members of the order of *Ca*. Brocadiales, but were not affiliated with either of the two established anammox families (i.e., *Ca*. Brocadiaceae and *Ca*. Scalinduaceae). Phylogenetic analysis of 16S rRNA gene sequences of lineages from the entire Planctomycetes phylum indicated that these three OTUs fell into the basal branch in the order *Ca*. Brocadiales, representing a novel family (Fig. 1A). This family was named GWA2-50-13 in the SILVA 138.1 release [25]. Close relatives of these three OTUs were also detected in marine sediments of the South China Sea and Bohai Sea [26–28], while sequences from terrestrial habitats especially groundwater [29, 30] are also included within this family (Fig. 1A).

To further elucidate the identity and potential ecological functions of this bacterial family, we recovered a metagenome-assembled genome (MAG), Bin_158, from the existing metagenome sequencing data of core GC08 [20]. The genome size of this MAG is 2.2 Mbp, with 2368 genes distributing in 623 scaffolds. It is estimated to be of 74% completeness with 3.4% redundancy (Table S1). Phylogenomic analysis based on the concatenated 120 single-copy genes of bacteria indicates that this MAG is a member of the family FULL-50-16-A (corresponds to the GWA2-50-13 family of the 16S rRNA gene phylogeny, Fig. 1B), a deep-branching family within the order *Ca*. Brocadiales. This classification is also supported by the phylogenomic analysis based on the concatenated 14 ribosomal proteins (Fig. S1). In addition to Bin_158 recovered from marine subsurface sediments, this family also contains nine MAGs from the groundwater environment, including RIFCSPLOWO2_02_FULL_50_16 (called FULL_50_16 thereafter), RIFCSPLOWO2_12_FULL_50_35 (FULL_50_35), RIFCSPHIGHO2_02_FULL_50_42 (FULL_50_42), RIFCSPHIGHO2_12_FULL_52_36 (FULL_52_36), GWA2_50_13, RIFCSPHIGHO2_12_FULL_51_37 (FULL_51_37), and RIFCSPHIGHO2_02_FULL_52_58 (FULL_52_58) independently recovered from different groundwater wells of Rifle, Colorado [30], and NC_groundwater_1467_Ag_S-0.65um_52_288 (52_288) and NC_groundwater_1822_Pr3_B-0.1um_52_21 (52_21) from groundwater of Modesto, North California [31]. Despite that no 16S rRNA gene can be identified in Bin_158, the 16S rRNA gene sequences of FULL_51_37, FULL_50_35, and FULL_52_36 were placed into the family GWA2-50-13 (Fig. 1A), suggesting congruency between the two phylogenetic trees and that the GWA2-50-13 family of the 16S rRNA gene phylogeny should correspond to the FULL-50-16-A family genomes (Fig. 1). The 10 MAGs of the FULL-50-16-A family can be divided into two genera: 2-02-FULL-50-16-A and 2-12-FULL-52-36. Average amino acid identity (AAI) analysis indicated that Bin_158 has 76% AAI with members of 2-02-FULL-50-16-A (Fig. S2), which is above the AAI threshold (>65%) proposed for genomes within the same genus [32] and therefore should represent a new species in this genus. This classification is also supported by the automatic classification of GTDB-tk [33]. To highlight the genetic potential of the anammox metabolism of these new lineages (see below), we propose to name the genus 2-02-FULL-50-16-A as *Candidatus* Bathyanammoxibius gen. nov. (bathy-; referring to the deep origins of the genomes in this family; anammoxi-, derived from Neo-Latin noun anammox, referring to the anammox potential; bius; referring to life in the Latin language). Accordingly, we also propose a new family name, *Candidatus* Bathyanammoxibiaceae fam. nov., for the FULL-50-16-A family to harbor the genus *Ca*. Bathyanammoxibius.

### Confined distribution and growth of *Ca*. Bathyanammoxibiaceae in the nitrate-ammonium transition zone

To explore the preferred niche(s) of *Ca*. Bathyanammoxibiaceae in marine sediments, we tracked its vertical distribution (represented by the sum of the three OTUs included in Fig. 1A) in the four AMOR sediment cores, based on the existing 16S rRNA gene amplicon sequencing data reported in [20] (the phylum level community structure of the total communities were shown in Fig. S4 therein). The relative abundances of *Ca*. Bathyanammoxibiaceae in the total communities of bacteria and archaea are higher than those of *Ca*. Scalinduaceae in cores GC04 and GC05 (Fig. 1I, L), but lower in cores GC08 and GC09 (Fig. 1C, F), with the relative abundance maxima (17% of the total communities in GC04 and 22% in GC05) detected within the NATZs (Fig. 1I, L). We also calculated the absolute abundances of *Ca*. Bathyanammoxibiaceae and *Ca*. Scalinduaceae in these cores by multiplying their relative abundances and the total cell numbers estimated from the qPCR quantification of 16S rRNA genes [20]. Similar to their relative abundances, the absolute abundances of these two families were highest within the NATZs (Fig. 1D, G, J, M). Based on reactive-transport modeling [20], the NATZ is where the anammox process is predicted to occur (Fig. 1E, H, K, N), and the confined distribution of *Ca*. Bathyanammoxibiaceae within the NATZs suggests that members of this family may be involved in the anammox process.

**Fig. 2:**
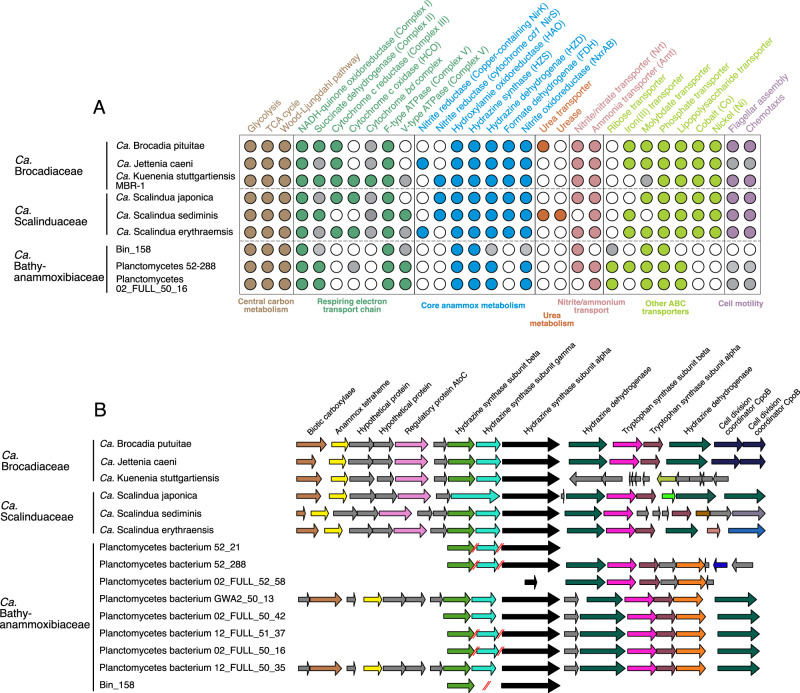
Metabolic features of *Ca*. Bathyanammoxibiaceae bacteria. **A** Comparison of metabolic potential of anammox bacteria from three different families. For each of the three anammox bacteria families, three genomes of high genome completeness were selected for the analysis. Filled circles represent the presence, grey circles denote the partial presence, and open circles indicate the absence of genes encoding the metabolic process. **B** Conservation of key genes encoding for the hydrazine metabolism in anammox bacteria genomes. The figure was produced in GeneSpy by comparing a region of 10 kb centered on the hydrazine synthase subunit alpha (HzsA). In addition to the three selected genomes from the families of Brocadiaceae and Scalinduaceae, all available genomes of the newly proposed family, *Ca*. Bathyanammoxibiaceae, were included in (**B**). The red double slash lines represent discontinuousness between contigs.

Considering that none of the *Ca*. Bathyanammoxibiaceae genomes contain genes responsible for bacterial flagellum synthesis (Fig. 2), *Ca*. Bathyanammoxibiaceae in the AMOR sediments are probably not able to migrate in the sediment pore space. The observed local subsurface peaks of *Ca*. Bathyanammoxibiaceae in the NATZs (Fig. 1D, G, J, M) represents another example of microbial in situ growth within energy-limited subseafloor sediments, adding to the growing body of such evidence previously reported for *Ca*. Scalindua [20], Thaumarchaeota [34, 35], and Atribacteria [36] in deep-sea sediments and various microbial taxa in estuarine sediments [37].

### Members of *Ca*. Bathyanammoxibiaceae have the anammox capacity

In accordance with their confined distribution in the anammox zone, genome annotation indicates that members of *Ca*. Bathyanammoxibiaceae are capable of performing anammox, as they have the core genetic machinery for the anammox metabolism (Fig. 2A): hydrazine synthase for converting NO and NH_4_^+^ to hydrazine, hydrazine dehydrogenase for hydrazine oxidation to dinitrogen gas (N_2_), and nitrite oxidoreductase for nitrite oxidation [38]. The gene cluster encoding the most critical enzyme of anammox, hydrazine synthase, is conserved in eight of the ten *Ca*. Bathyanammoxibiaceae genomes (Fig. 2B), whereas its absence in the remaining two genomes (only a partial hydrazine synthase alpha subunit exists in FULL_52_58 and none of the subunits is present in FULL_52_36) can be attributed to the low genome completion levels (<73% complete; Table S1).

*Ca*. Bathyanammoxibiaceae genomes contain hydrazine synthases-encoding genes that are not only of similar gene arrangement but also similar structure to those of characterized anammox bacteria. *Ca*. Bathyanammoxibiaceae genomes encode the hydrazine synthase (HZS, constituted by the alpha, beta, and gamma subunits; Fig. 2B) as characterized anammox bacteria in the families *Ca*. Brocadiaceae and *Ca*. Scalinduaceae, except for *S. brodae*, *S. profunda*, and *S. japonica* in which the beta and gamma subunits are fused into a single polypeptide [39, 40]. Bin_158 recovered from AMOR sediments, has genes encoding only the hydrazine synthase subunits alpha (HzsA) and gamma (HzsC) on two separated contigs, probably due to the incomplete nature of this genome. Furthermore, the two heme-binding motifs (CXXCH) in the HZS alpha subunit for electron transfer and the 15-amino-acid loop in the HZS beta subunit that directs the trafficking of substrates and products of the alpha and gamma subunits, are also conserved in the *Ca*. Bathyanammoxibiaceae genomes (Fig. S3). These analyses strongly suggest that *Ca*. Bathyanammoxibiaceae have the hydrazine synthesis capacity, the most critical step of the anammox metabolism.

Phylogenetic analysis of the alpha (*HzsA*), beta (*HzsB*), and gamma (*HzsC*) subunits of the hydrazine synthase indicated that *Ca*. Bathyanammoxibiaceae formed a separate, basal branch from the families *Ca*. Brocadiceae and *Ca*. Scalinduaceae (Fig. S4). These three phylogenetic trees show similar topologies and are congruent with the above-described ones based on 16 rRNA gene, ribosomal proteins, and 120 single-copy genes (Fig. 1A, B), implying that anammox bacteria acquired this critical enzyme by vertical inheritance from the common ancestor of anammox bacteria rather than by horizontal gene transfer events.

Nitrite reduction responsible for the generation of nitric oxide from nitrite is also part of the core anammox metabolism [38], and can be carried out by the copper-containing nitrite reductase (NirK) or the cytochrome *cd1*-containing nitrite reductase (NirS). Members of *Ca*. Bathyanammoxibiaceae lack both of these two nitrite reductases (Fig. 2A), similar to some anammox bacteria in the genus of *Ca*. Brocadia [41]. However, recently Ferousi et al [42] revealed that anammox bacteria may also use a specific hydroxylamine oxidoreductase (HAO) (Kustc0457/458 in *Kuenenia stuttgartiensis* or HAO2 following the definition of [41]) to perform the nitrite reduction process. While *Ca*. Bathyanammoxibiaceae genomes do not encode HAO2, they encode 5–7 variants of HAO including 1–3 variants of HAO7 (Fig. S5). The branch of these HAO7 variants is the closest neighbor to HAO2 and share 47–53% amino acid identities with Kustc0457/458 (Fig. S5), but to what extent this allow *Ca*. Bathyanammoxibiaceae to have the nitrite reduction capacity is currently unknown.

Similar to the characterized anammox bacteria in the families *Ca*. Brocadiaceae and *Ca*. Scalinduaceae, members of *Ca*. Bathyanammoxibiaceae also encode the nitrite oxidoreductase (NXR), which in anammox bacteria is an essential enzyme with a tubular architecture functioning in the anammoxosome organelle [43]. Phylogenetic analysis of the nitrite oxidoreductase alpha subunit (NxrA) showed that members of *Ca*. Bathyanammoxibiaceae formed a separated cluster from characterized anammox bacteria in the families *Ca*.Brocadiaceae and *Ca*. Scalinduaceae, the latter formed a big cluster together with nitrite-oxidizing *Nitrospira* and *Nitrospina* and also some other diverse bacteria (Fig. S6). Again, this phylogenetic branching pattern among the three anammox bacteria families suggested that they gained this key enzyme through vertical inheritance.

*Ca*. Bathyanammoxibiaceae genomes encode the same central carbon metabolism pathways as other characterized anammox genomes. These include the Wood-Ljungdahl pathway (Fig. 2A), which could enable them to fix CO_2_ to generate acetyl-CoA and pyruvate, using electrons derived from the hydrazine oxidation [38, 44, 45]. Also included are the tricarboxylic acid (TCA) cycle and the gluconeogenesis pathway (Fig. 2A), which, respectively, can use acetyl-CoA and pyruvate produced from the Wood-Ljungdahl pathway to synthesize biomass precursors like amino acids and carbohydrates [44]. In addition, some *Ca*. Bathyanammoxibiaceae genomes contain genes for the ABC transporter of ribose (Fig. 2A), which could indicate that these bacteria can assimilate small carbohydrates using electrons derived from the hydrazine degradation. Similar to *Ca*. Scalindua sediminis, the three representative genomes of *Ca*. Bathyanammoxibiaceae encode both the F-type ATPase and V-type ATPase (Fig. 2A), which could be integrated into the two different ATP generation processes in these genomes. This may represent an adaptation strategy for members of *Ca*. Bathyanammoxibiaceae thrive in the energy-limiting subsurface.

Because they broadly occupy the same niche of *Ca*. Scalinduaceae, the NATZs, *Ca*. Bathyanammoxibiaceae may need surrounding denitrifying bacteria to provide them nitrite, one of the direct substrates of anammox bacteria [46], as previously proposed for *Ca*. Scalindua sediminis [20]. The ABC transporters for iron (Fe^3+^) and molybdate (MoO_4_^2−^) seem to be conserved in all anammox genomes (Fig. 2A), which may be critical for these putative anammox bacteria in incorporating these metals as the co-factor of heme-containing cytochromes and molybdate-containing enzymes that are critical and widespread proteins in anammox bacteria [47]. Unlike *Ca*. Scalindua sediminis [20], members of *Ca*. Bathyanammoxibiaceae do not encode urease or cyanase, suggesting that they may not be capable of utilizing these alternative substrates.

### A wide distribution of *Ca*. Bathyanammoxibiaceae in both marine and terrestrial environments

**Fig. 3:**
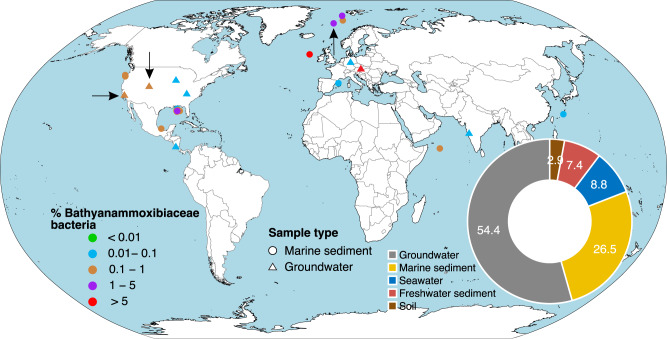
Global occurrence of *Ca*. Bathyanammoxibiaceae bacteria. The occurrence and relative abundances of *Ca*. Bathyanammoxibiaceae were determined in the IMNGS database using the 16S rRNA gene sequences of GWA2_50_13 and FULL_52_36 as the queries. Sequences (>200 bp) with similarity higher than 95% to the queries were regarded as positive hits. At most of the sites, *Ca*. Bathyanammoxibiaceae was detected in multiple depths and the highest relative abundances are reported. The three sites where the available *Ca*. Bathyanammoxibiaceae MAGs originate from are indicated by black arrows. The insert pie chat shows the environmental distribution of the 16S rRNA gene sequences included in the GWA2-50-13 family in the SILVA 138.1 Release.

*Ca*. Bathyanammoxibiaceae is present in various ecosystems. Through the search against the available high-throughput sequencing data in IMNGS [48], we detected the presence of *Ca*. Bathyanammoxibiaceae in a total of 128 samples (Fig. 3). For marine sediments, in addition to AMOR sediments [e.g., [49] and Fig. 1], *Ca*. Bathyanammoxibiaceae account for >1% of the total communities in the surface sediments of the Gulf of Mexico [50] and Rockall Bank [51], and >0.1% of the total communities in subsurface sediments of the Hydrate Ridge, and the Indian and Pacific Ocean (Fig. 3). Consistent with the 9 MAGs recovered from the groundwater environment, *Ca*. Bathyanammoxibiaceae were also detected in groundwater in the US, off the Danube Riverbank in Austria [52], Central Germany [53], and India (Fig. 3).

We also assessed the distribution of *Ca*. Bathyanammoxibiaceae by checking the origins of the 77 16S rRNA gene sequences included in the SILVA 138.1 Release, which were mainly derived from clone library-based studies. The majority of them were recovered from groundwater (54%) and marine sediment (26%) environments (Fig. 3), consistent with the dominance of groundwater and marine sediment as the habitat of *Ca*. Bathyanammoxibiaceae in this high-throughput sequencing data analysis. Sequences of *Ca*. Bathyanammoxibiaceae were also detected in coral reef carbonate sediments off Hawaii [54], South China Sea sediments [55], and Bohai Sea sediments [28]. In addition, we noticed that 9% of the sequences were from seawater (e.g., Deep brine pool in the Red Sea [56]), 7% from freshwater sediments, and 3% from soils (Fig. 3). These results suggested that *Ca*. Bathyanammoxibiaceae bacteria are potentially important players in the nitrogen cycle in a number of marine and terrestrial environments.

### Conclusion and Outlook

Through genomic recovery and characterization, we report a new family of bacteria within the order of *Ca*. Brocadiales that have the genetic potential for the anammox metabolism. By geochemical characterization and ecological surveys, our study also provides valuable guidance (e.g., ideal inoculum and cultivation conditions) for future enrichment and cultivation efforts, which are critical to confirm the predicted function and activity of the bacteria in this family. When their anammox activity got confirmed, the genomes in the deeply branching *Ca*. Bathyanammoxibiaceae family may be valuable to delineate the evolutionary history of anammox in the nitrogen cycle [57]. Importantly, *Ca*. Bathyanammoxibiaceae contains members from both the marine and terrestrial environments, contrasting the partitioning between the previously recognized anammox families of *Ca*. Scalinduaceae and *Ca*. Brocadiaceae, and therefore represents an ancient lineage that may preserve some features of the anammox bacteria before their radiation to the marine and terrestrial environments.

In addition to the absence of the anammox function proof, there are also other prominent open questions pertaining to this family of bacteria. How are they competing (or cooperating) with the co-existing *Ca*. Scalinduaceae in the NATZ of marine sediments? Why are they apparently so widespread in the energy-limited subsurface? Addressing these questions and adding *Ca*. Bathyanammoxibiaceae to the important functional guild anammox may lead to a better understanding about the biogeochemical impacts and evolutionary history of anammox bacteria on Earth.

## Materials and methods

### Sampling collection and characterization

This study uses samples and data generated and reported in [20], in which the procedures of sample collection, processing, and data generation were thoroughly described. Briefly, the four sediment cores (2.0–3.6 m long) were retrieved by gravity coring from the seabed of ~2000 of water depth on the ridge flanks of the Arctic Mid-Ocean Ridge beneath the Norwegian-Greenland Sea. The retrieved cores were split to two halves (sampling and archiving halves), and the oxygen measurement (using a needle-type fiber-optic oxygen microsensor (optodes, PreSens)) and the subsampling of microbiology samples (using sterile 10-mL cutoff syringes) and porewater extraction were performed immediately on the sampling half using Rhizons samplers after the split. Nitrate, nitrite, and ammonium concentrations in the porewater were measured colorimetrically by a Quaatro 114 continuous flow analyzer (SEAL Analytical Ltd, Southampton, UK).

In each core, a downward flux of nitrate and an upward flux of ammonium were observed to be co-consumed in the middle part of the retrieved cores, which was named nitrate-ammonium transition zone (NATZ) [20]. The occurrence and rates of the anammox process in the NATZs of these cores were predicted using a reaction-transport model, which considers six reactions of carbon, nitrogen, oxygen, and manganese transformation during the early diagenesis of organic matter in marine sediments to simulate the measured porewater profiles of O_2_, NO_3_^−^, NH_4_^+^, dissolved inorganic carbon, and total organic carbon contents [20].

### 16S rRNA gene amplicon preparation, sequencing, and analysis

Total DNA in the sediment samples was extracted using the PowerLyze DNA extraction kits (MOBIO Laboratories, Inc.). Amplicon of the 16S rRNA gene were prepared using a two-round PCR amplification strategy with the “universal” primers of 515 F/806r [20]. The amplicon libraries were sequenced on an Ion Torrent Personal Genome Machine. As described in [20], sequencing reads were quality filtered and trimmed to 220 bp using the USEARCH v11.0.667 pipeline [58]. The taxonomic classification of OTUs was performed using the lowest common ancestor algorithm implemented in the python version of CREST4 (the latest version of CREST [59]) against the SILVA 138.1 Release [25]. This study focused on three OTUs that were classified as members of the order *Ca*. Brocadiales, but were not affiliated with either of known anammox families: *Ca*. Brocadiaceae and *Ca*. Scalinduaceae.

### Genome binning and refinement

Metagenome sequencing data were generated and reported in [20]. The detailed procedures of the DNA extraction, library preparation, metagenome sequencing, raw data quality control, assembly, genome binning and refinement were also detailed therein [20]. Briefly, DNA were extracted from ~7 g sediment (0.7 g sediment in 10 individual lysis tubes) of four depths in core GC08. Metagenomic libraries were sequenced (2 × 150 bp paired-end) by an Illumina HiSeq 2500 sequencer at the Vienna Biocenter Core Facilities GmbH (Vienna, Austria). The quality of the raw sequencing data were first checked using FastQc v0.11.9 [60], and adapters were accordingly removed and reads were trimming based on the quality scores using Trimmomatic v0.39 [61]. The quality-controlled paired-end reads were de novo assembled into contigs using MEGAHIT v1.1.2 [62] with the k-mer length varying from 27 to 117. Contigs larger than 1000 bp were automatically grouped into genome bins using MaxBin2 v2.2.5 [63] with the default parameters. The quality of the obtained genome bins was assessed using the option “lineage_wf” of CheckM v.1.0.7 [64]. Genome bins of >50% completeness were manually refined using the tool gbtools v2.6.0 [65] based on the GC content, taxonomic assignments, and differential coverages of contigs across multiple samples. To generate the input data of the genome refinement, coverages of contigs in each sample were determined by mapping trimmed reads onto the contigs using BBMap v.37.61 [66]. Taxonomy classification of contigs were assigned by BLASTn [67] according to the taxonomy of the single-copy marker genes in contigs. SSU rRNA sequences in contigs were identified using Barrnap [68] and classified using VSEARCH (56) with the SILVA 132 release (57) as the reference. All refined MAGs were classified using GTDB-tk v1.5.0 [33] with the default setting. Only MAGs of ≥70% completeness with <5% redundancy were included for downstream analyses.

In this study, Bin_158 was found to be affiliated with a family without known anammox genera and was therefore subject to further analysis. To improve the quality of the genome of Bin_158, quality-trimmed reads of the sample GC08_160cm within the NATZ, were mapped onto the contigs using BBmap [66], and the successfully aligned reads were re-assembled using SPAdes v3.12.0 [69] with the k-mers of 21, 33, 55, and 77. After removal of contigs shorter than 1000 bp, the resulting scaffolds were visualized and manually re-binned using gbtools v2.6.0 [65] as described above. The quality of the resulting Bin_158 genome was checked using the CheckM v1.0.7 “lineage_wf” command again, based on the Planctomycetes marker gene set.

### Genome annotation

Bin_158 were annotated together with the MAGs included in the family FULL-50-16-A in the GTDB Release 06-RS202 (https://gtdb.ecogenomic.org/). In addition, two closely relative MAGs not then included in the GTDB database, e.g., NC_groundwater_1467_Ag_S-0.65um_52_288 (52_288) and NC_groundwater_1822_Pr3_B-0.1um_52_21 (52_21) reported in [31], were also included in the genome annotation for comparison. Genes in these genomes were predicted using Prodigal [70]. Genome annotation was conducted using Prokka v1.13 [71], eggNOG [72], and also BlastKoala [73] using the KEGG database. The functional assignments of genes of interest were also confirmed using BLASTp [68] against the NCBI RefSeq database. The metabolic pathways were reconstructed using KEGG Mapper [74]. The gene arrangement around the hydrazine synthase genes were visualized using GeneSpy v1.2 [75], with the GFF files produced by Prokka as the input.

### Phylogenetic analyses

To pinpoint the phylogenetic placement of Bin_158 and the relative genomes in the family FULL-50-16-A, we performed phylogenetic analyses for them together with high-quality genomes of the Planctomycetes phylum that was included in the GTDB Release 06-RS202. The 120 single-copy genes were identified, aligned, and concatenated using GTDB-tk v1.5.0 with the “classify_wf” command. The maximum-likelihood phylogenetic tree was inferred based on this alignment using IQ-TREE v1.5.5 [76] with LG + F + R7 the best-fit model selected by ModelFinder [77], and 1000 ultrafast bootstrap iterations using UFBoot2 [78]. To provide support to this phylogenomic analysis, we also performed the phylogenomic analysis based on the 14 syntenic ribosomal proteins (rpL2, 3, 4, 5, 6, 14, 16, 18, 22, and rpS3, 8, 10, 17, 19) that have been demonstrated to undergo limited lateral gene transfer [79]. These selected proteins were identified in Anvi’o v7.1 (67) using Hidden Markov Model (HMM) profiles and aligned individually using MUSCLE [80]. Alignment gaps were removed using trimAl [81] with the mode of “automated”. Individual alignments of ribosomal proteins were concatenated. The maximal likelihood phylogenetic tree was reconstructed using IQ-TREE v1.5.5 [76] with LG + R7 as the best-fit model.

A maximum-likelihood phylogenetic tree based on 16S rRNA genes was also constructed to highlight to the phylogenetic placement of the *Ca*. Bathyanammoxibiaceae family in the Planctomycetes phylum. To expand this family on the tree beyond the available genomes, the three *Ca*. Bathyanammoxibiaceae OTUs from the amplicon sequencing and their close relatives identified via BLASTn [82] in the NCBI database were also included. Sequences were aligned using MAFFT-LINSi [83] and the maximum-likelihood phylogenetic tree was inferred using IQ-TREE v1.5.5 [76] with GTR + F + R3 as the best-fit substitution model and 1000 ultrafast bootstraps, following the procedure described above.

For the phylogeny of HzsA (hydrazine synthase subunit alpha), the genomes of known anammox bacteria were downloaded from the NCBI database, annotated using Prokka v1.13 [71], and the HzsA amino acid sequences were extracted. Additional HzsA sequences of uncultured anammox deposited in the NCBI database were also identified using BLASTp [67] using the HzsA sequences of *Ca*. S. sediminis and Bin_158 as the query and the *E*-value of 10^−6^. Sequences were aligned using MAFFT-LINSi [83] and the maximum likelihood phylogenetic tree was inferred using IQ-TREE v1.5.5 following the procedure described above.

For the phylogeny of NxrA (nitrite oxidoreductase alpha subunit), the sequence of Bin_158 was used as the query in the BLASTp [82] search in the NCBI database (>50% similarity and *E*-value of 10^−6^) to identify its close relatives. These sequences were aligned using MAFF-LINSi [83] with reference sequences from ref. [84], and complemented with known nitrite-oxidizing bacteria. The alignment was then trimmed using trimAl [81] with the mode of “automated”. Maximum likelihood phylogenetic trees were reconstructed using IQ-TREE v1.5.5 [76] with the LG + C20 + F + G substitution model and 1,000 ultrafast bootstraps.

### Global occurrence of *Ca*. Bathyanammoxibiaceae

We used the 16S rRNA gene sequence of Planctomycetes bacteria GWA2_50_13 and FULL_52_36 as the queries in the IMNGS (Integrated Microbial Next Generation Sequencing, https://www.imngs.org/) database [48] to explore the global occurrence of *Ca*. Bathyanammoxibiaceae. The similarity threshold was set at 95%, because (1) the inter-families identities in the order of Brocadiales were determined to be around 90%, and (2) generally species in the same families were suggested to share a 16S rRNA gene identity of 92% [32]. The sequence length threshold was set at 200 bp, because most of the sequences in the IMNGS database are short reads rather than full-length 16S rRNA gene sequences. Only hits with >5 sequences and >0.001 relative abundance were considered. To assess the distribution range of *Ca*. Bathyanammoxibiaceae based on sequence survey data prior to the emergence of Next Generation sequencing, we also parsed the information of the high-quality 16S rRNA sequences (*n* = 77) included in the GWA2-50-13 family of the SILVA 138.1 Release, and grouped the sample origins into the following categories: groundwater, marine sediment, seawater, freshwater sediment, and soil.

## Supplementary information

Supplementary Figures

Supplementary Table S1

## Data Availability

All sequencing data used in this study are available in the NCBI Short Reads Archive under the project number PRJNA529480. In particular, raw metagenomic sequencing data are available in the NCBI database under the BioSample number SAMN11268106. The genome sequence of Bin_158 is available under the accession number JAFNKF000000000. All other genomes analyzed in this study are available in the NCBI database under the accession numbers listed in Table S1.
