# Supplementary material for: Introducing Candidatus Bathyanammoxibiaceae, a family of bacteria with the anammox potential present in both marine and terrestrial environments
Source: ISME Commun. 2022 May 19;2:42. doi: 10.1038/s43705-022-00125-4 (PMC9723696; doi:10.1038/s43705-022-00125-4)
Supplement: Supplementary file 1 — Supplementary Figures [file 43705_2022_125_MOESM1_ESM.docx]

**Supplementary Information**


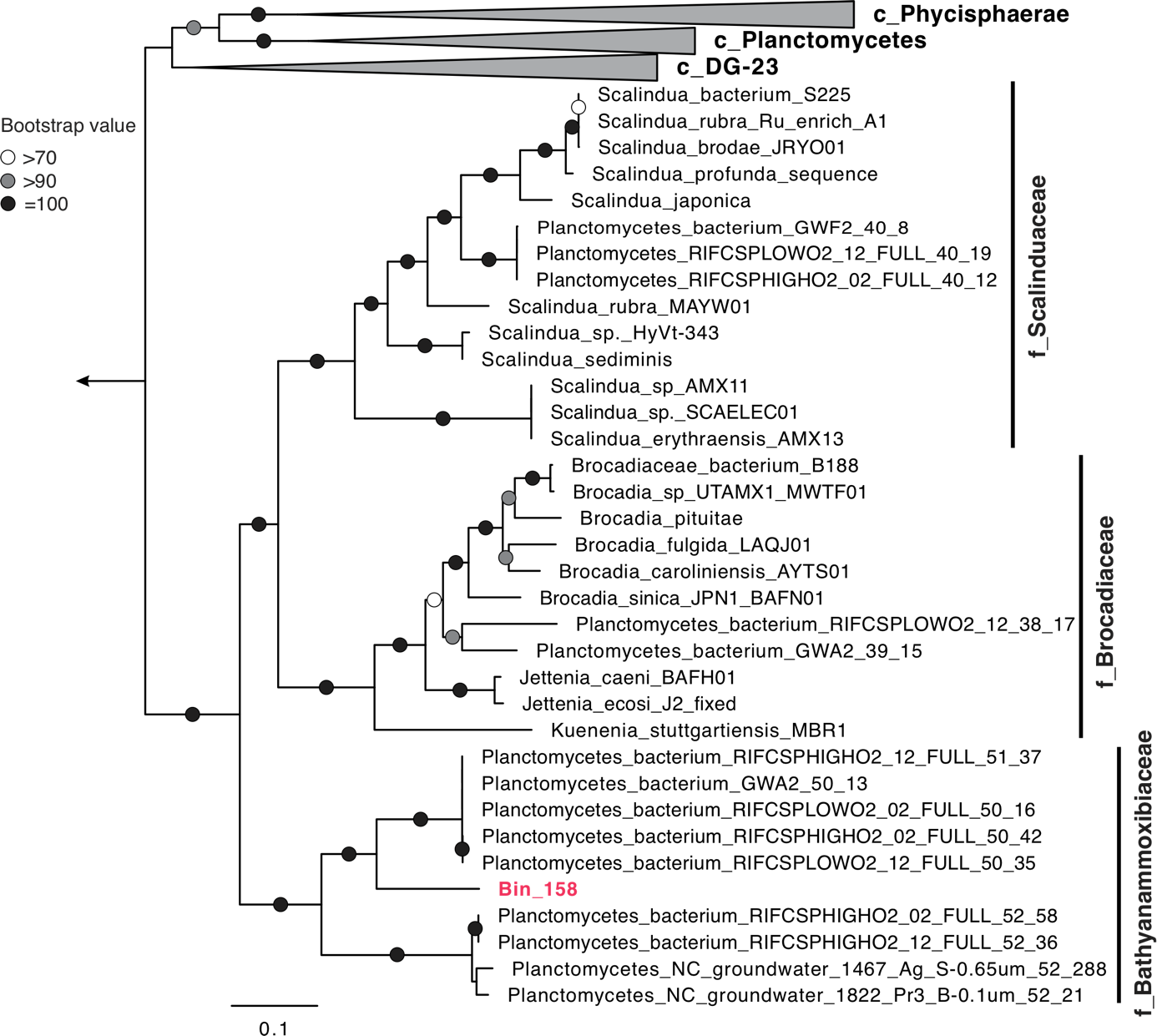


**Fig. S1. Maximum-likelihood phylogenetic tree of anammox bacteria in the order of Brocadiales, based on the concatenated 14 ribosomal proteins.** This tree was reconstructed for selected genomes from all major classes within the Planctomycetes phylum, and only branches of the order of Brocadiales (i.e., the three families of anammox bacteria) are expanded. Bootstrap values of >70 are shown with symbols listed in the legend. The scale bar shows estimated sequence substitutions per residue.

**
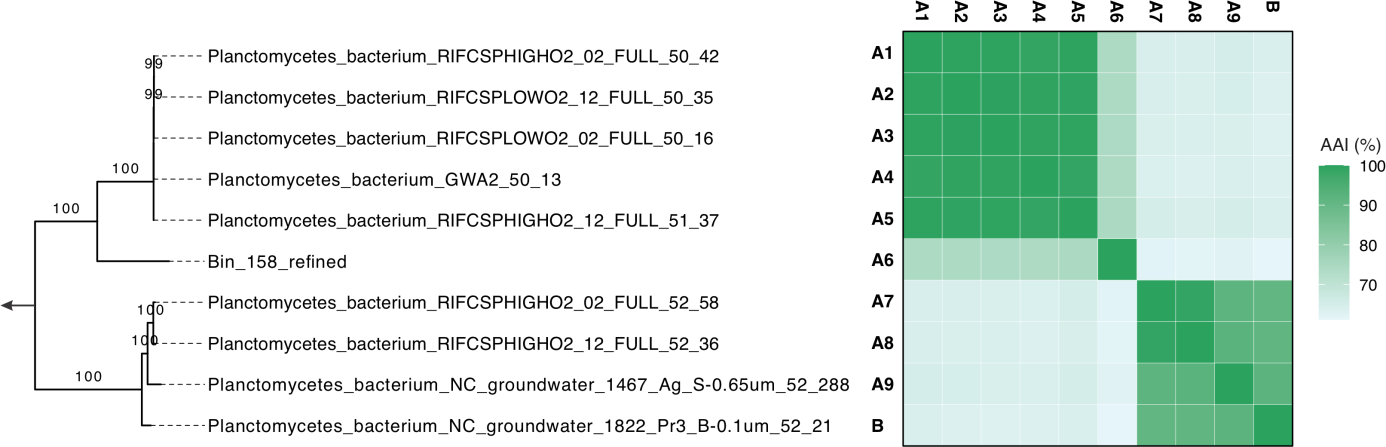
**

**Fig. S2. Average amino acid identity (AAI) between genomes in the family of *Candidatus* Bathyanammoxibiaceae.** AAIs were determined using CompareM (https://github.com/dparks1134/CompareM) using the default parameters. The phylogenetic tree is the same as that in Fig. 1B.


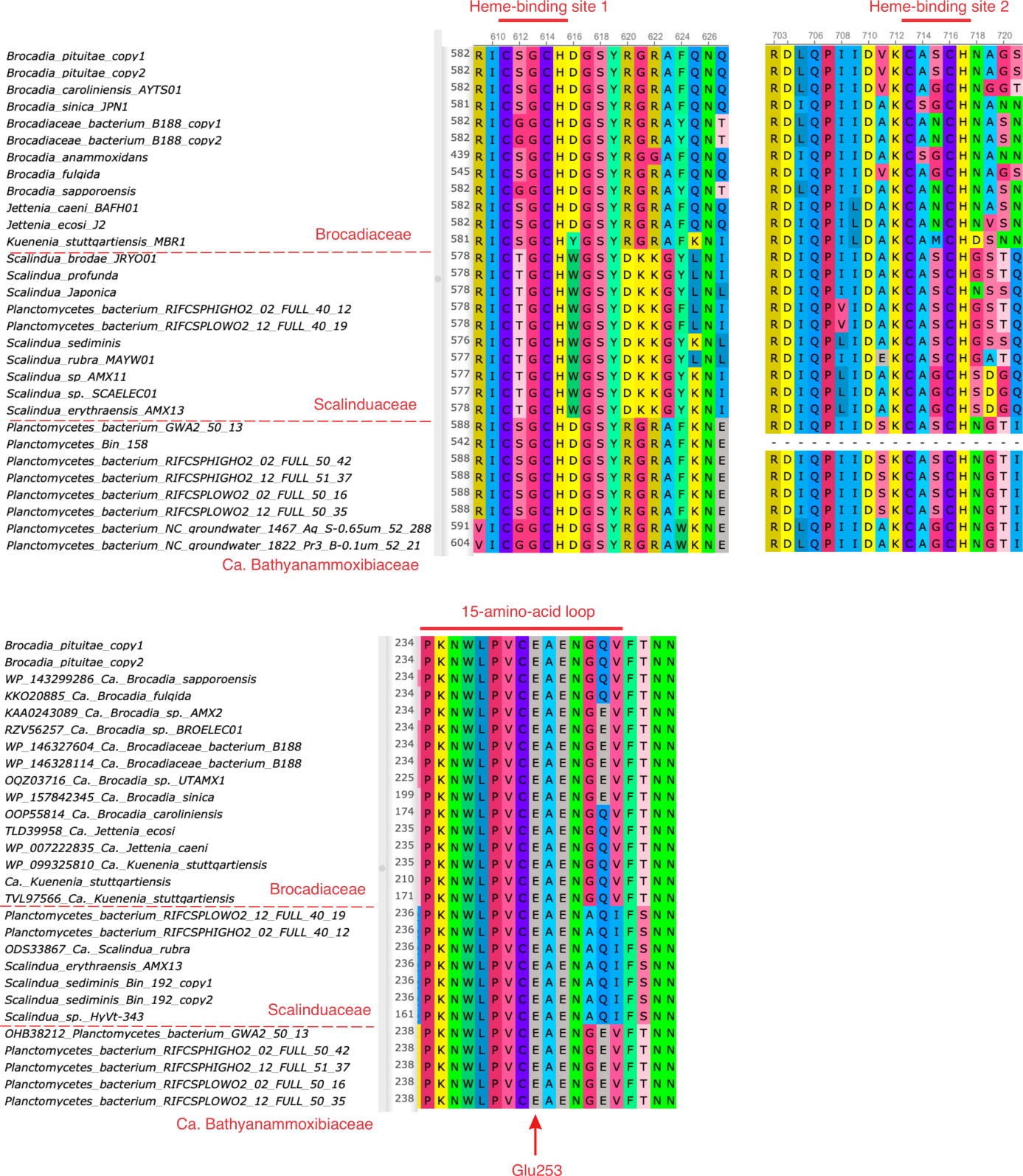


**Fig. S3. Amino acid sequences alignments showing the conservation of key features of hydrazine synthase in Ca. Bathyanammoxibiaceae genomes. (A)** Sequence alignment of hydrazine synthase alpha subunit of anammox bacteria highlighting the two heme-binding motifs (CXXCH). (**B**) Sequence alignment of hydrazine synthase beta subunit of anammox bacteria highlighting the 15-amino-acid loop.

**
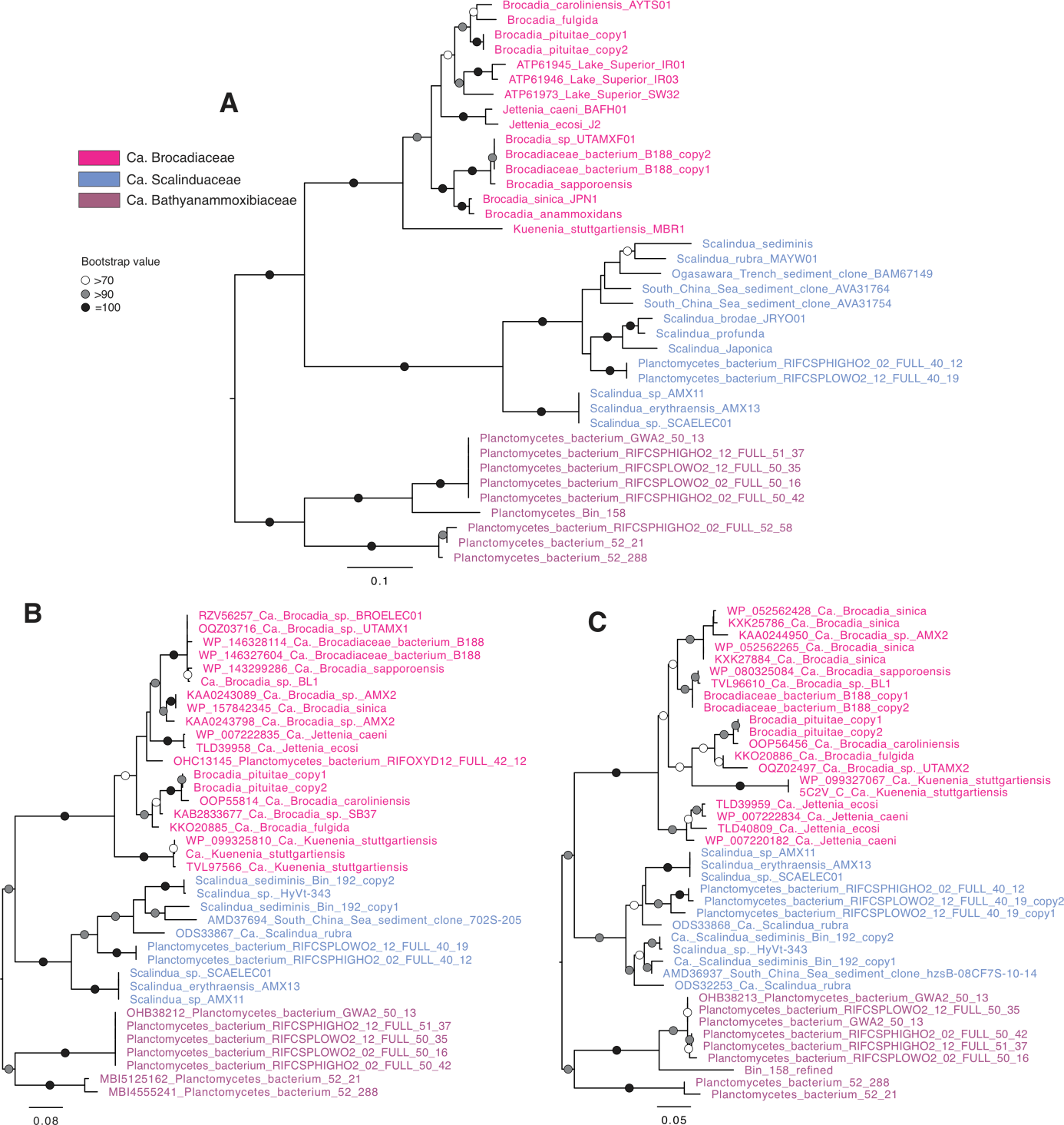
**

**Fig. S4. Maximum-likelihood phylogenies of the hydrazine synthase alpha (A), beta (B), and gamma (C) subunits of anammox bacteria.** All phylogenetic trees were reconstructed using IQ-TREE with 1,000 ultrafast bootstrap iterations. Sequences of the three anammox families are highlighted in different colors. Bootstrap values of >70 are shown with symbols listed in the legend. The scale bars show estimated sequence substitutions per residue.

**
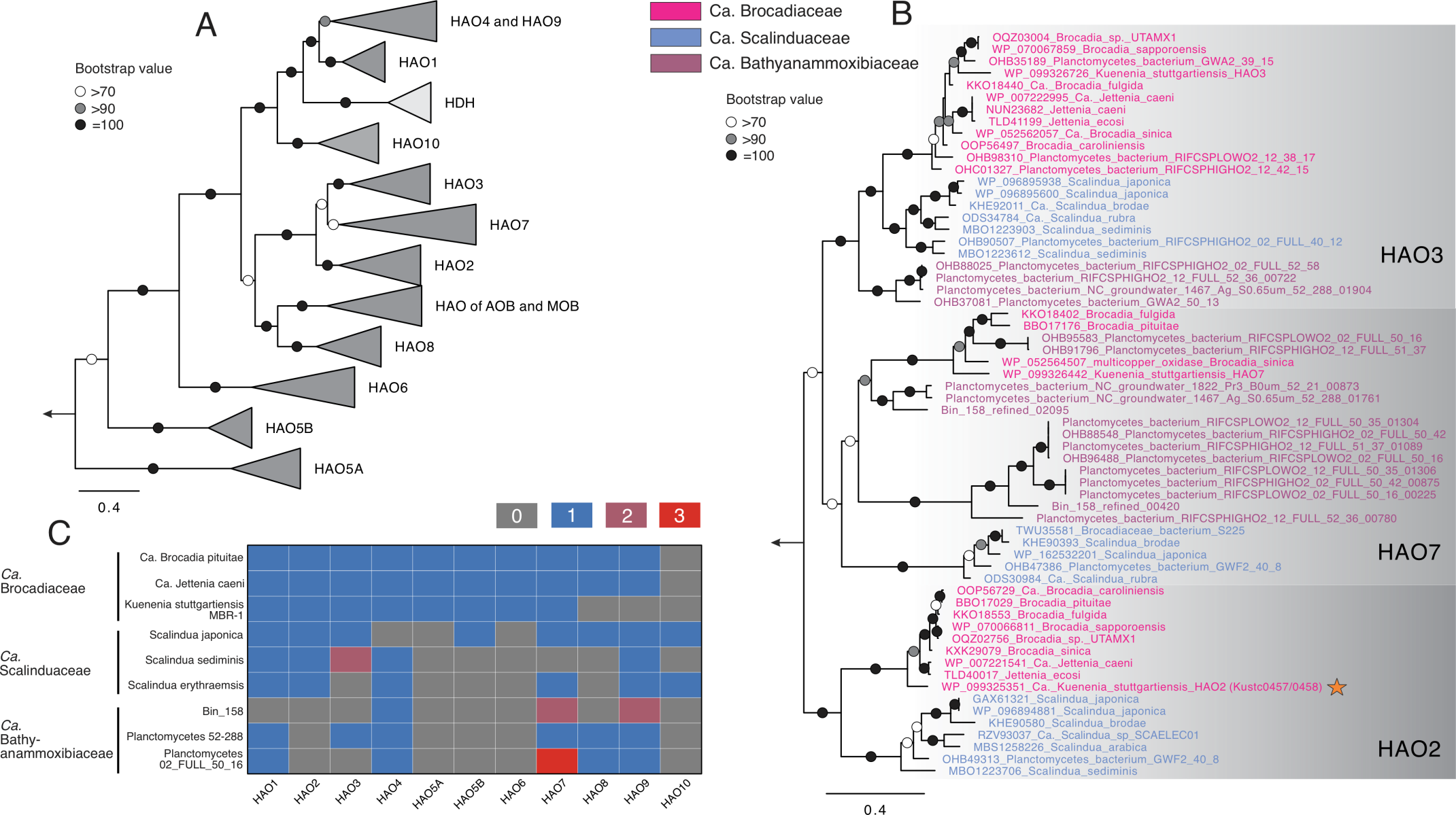
**

**Fig. S5. Phylogeny and occurrence of hydroxylamine oxidoreductase (HAO) in anammox bacteria. (A)** Maximum-likelihood phylogenetic tree of anammox HAO inferred using IQ-TREE with LG+R7 as the best-fit evolutionary model and 1,000 ultrafast bootstrap iterations. Note that anammox HAO is homologues to HAO of ammonia- and methane-oxidizing bacteria and hydrazine dehydrogenase (HDH) of anammox bacteria. The clades were named following [1]. **(B)** Expanded phylogenetic tree showing the clades of HAO2 (including the sole characterized nitrite reducing HAO in *Ca.* Kuenenia stuttgartiensis [2]), HAO3, and HAO7. **(C)** The occurrence of different hydroxylamine oxidoreductases in representative genomes of the three anammox bacteria families.

**
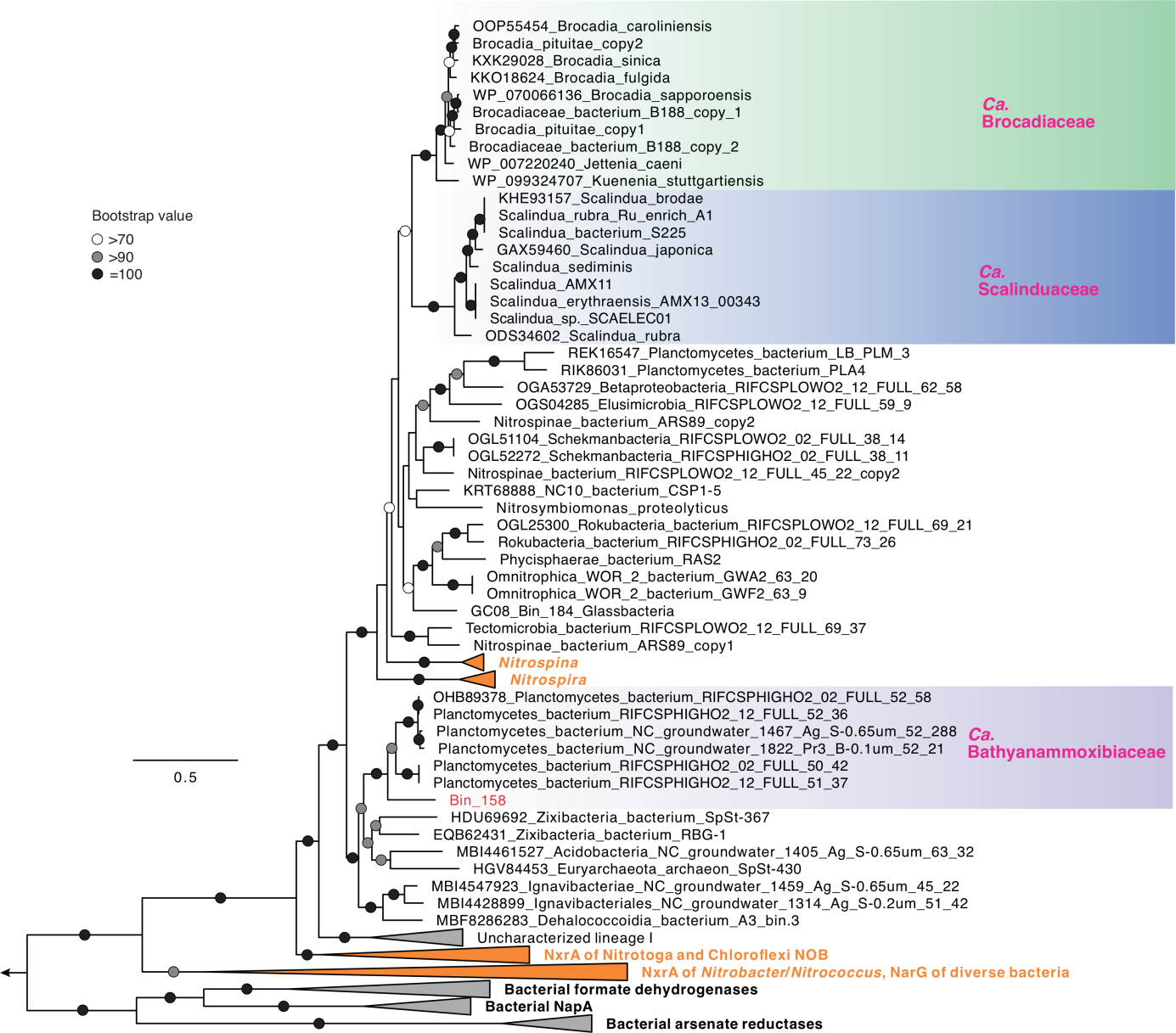
**

**Fig. S6. Maximum-likelihood phylogenies of the nitrite oxidoreductase alpha subunit of anammox bacteria.** The phylogenetic tree was reconstructed using IQ-TREE with LG+R9 as the best-fit evolutionary model and 1000 ultrafast bootstraps. Bin_158 recovered in this study is highlighted in red. Lineages containing characterized nitrite-oxidizing bacteria are highlighted in orange. The lineages of the three families of Brocadiales are shown using the same colors of boxes as Fig. 2B. Bootstrap values of >70 are shown with symbols listed in the legend. The scale bars show estimated sequence substitutions per residue.

**References**

1. Okubo T, Toyoda A, Fukuhara K, Uchiyama I, Harigaya Y, Kuroiwa M et al. The physiological potential of anammox bacteria as revealed by their core genome structure. DNA Research. 2021; 28:dsaa028.

2. Ferousi C, Schmitz RA, Maalcke WJ, Lindhoud S, Versantvoort W, Jetten MSM et al. Characterization of a nitrite-reducing octaheme hydroxylamine oxidoreductase that lacks the tyrosine cross-link. Journal of Biological Chemistry. 2021; 296:100476.
